# Current Management of Neuroendocrine Tumour Liver Metastases

**DOI:** 10.1007/s11912-024-01559-w

**Published:** 2024-06-13

**Authors:** Aditya Sharma, Mughilan Muralitharan, John Ramage, Dominique Clement, Krishna Menon, Parthi Srinivasan, Mohamed Elmasry, Nick Reed, Matthew Seager, Rajaventhan Srirajaskanthan

**Affiliations:** 1 Department of Gastroenterology, King’s College Hospital, SE5 9RS London, U.K.; 2 Neuroendocrine Tumour Unit, Institute of Liver Studies, King’s College Hospital, SE5 9RS London, U.K.; 3 Institute of Liver Studies, King’s College Hospital, SE5 9RS London, U.K.; 4 Department of Oncology, Beatson Centre, G12 0YN Glasgow, U.K.; 5 Department of Radiology, King’s College Hospital, SE5 9RS London, U.K.; 6 Neuroendocrine Tumour Unit Institute of liver studies, King’s College Hospital, SE5 9RS London, U.K.

**Keywords:** Neuroendocrine Tumour, Liver Metastases, PET Imaging, MRI Scans, Liver Resection, Embolisation, Liver Transplantation

## Abstract

**Purpose of review:**

This article aims to illustrate the current state of investigations and management of liver metastases in patients with Neuroendocrine Neoplasms. Neuroendocrine tumours (NETs) are rising in incidence globally and have become the second most prevalent gastrointestinal malignancy in UK and USA. Frequently, patients have metastatic disease at time of presentation. The liver is the most common site of metastases for gastro-enteropancreatic NETs. Characterisation of liver metastases with imaging is important to ensure disease is not under-staged.

**Recent Findings:**

Magnetic resonance imaging and positron emission tomography are now becoming standard of care for imaging liver metastases. There is an increasing armamentarium of therapies available for management of NETs and loco-regional therapy for liver metastases. The data supporting surgical and loco-regional therapy is reviewed with focus on role of liver transplantation.

**Summary:**

It is important to use appropriate imaging and classification of NET liver metastases. It is key that decisions regarding approach to treatment is undertaken in a multidisciplinary team and that individualised approaches are considered for management of patients with metastatic NETs.

## Introduction

Neuroendocrine neoplasms (NENs) are a heterogenous group of tumours that most commonly originate in the gastroenteropancreatic tract (GEP) [1]. The GEP NENs display a propensity to metastasize to the liver [1, 2]. NENs are subdivided into neuroendocrine tumours (NETs) and neuroendocrine carcinomas (NECs) based on the tumour morphology. NETs are well differentiated, whereas NECs are poorly differentiated and exhibit aggressive behaviour leading to poorer outcomes [2, 3]. The grade of NETs is determined by mitotic count or by Ki-67 index [4]. Grade 1 NETs have a Ki-67 of < 3% and mitotic count 0–2 per high powered field (HPF), Grade 2 NETs have a Ki-67 of 3-20% with mitotic count of 3–20, and Grade 3 NETs have a Ki-67 of > 20% and mitotic count > 20 per HPF. NETs characteristically display abundant expression of somatostatin receptors (SSTRs) primarily SSTR-2 and SSTR-5 [5]. Functional neuroendocrine tumours can secrete bioactive substances, including serotonin, which can lead to carcinoid syndrome [6]. Carcinoid syndrome manifests with cutaneous flushing, diarrhoea, and cardiac valvular abnormalities [6].

Liver metastases frequently occur in patients with GEP NETs. The prevalence from studies ranges from 21 to 90%, with 12-74% having synchronous liver metastases [3, 7–11]. Approximately 50% of patients with pancreatic primaries and 60 to 75% of patients with small bowel NETs present with liver metastases synchronously or develop metachronous liver metastases [12, 13]. Liver metastases are much less common when primary tumours arise from the stomach, appendix, and other gastrointestinal sites.

The presence of neuroendocrine tumour liver metastases (NELMs) is a prognostic factor in survival of patients with NETs, regardless of primary tumour location [14]. Patients with localised disease often have a 10-year survival of greater than 90%, however in patients with liver metastases the survival is much lower and reported to be between 10 and 75% at 5 years [1, 15–17]. Numerous publications have supported the role of surgical resection with lower debulking thresholds and considerations of liver transplantation (LT) as a possible management option [4].

Hence, management of NETs and NELMs is very important and requires careful consideration. This review article will highlight the characterisation of liver metastases and the imaging criteria that can help fully elucidate the extent of disease burden. Then, we will discuss the treatment options available for patients with NELMs.

## Imaging for NET in Liver Metastases

Characterisation of NELMs is based on imaging criteria. At time of initial staging most patients undergo a contrast enhanced computed tomography (CT) scan [2]. The recommendation from European Neuroendocrine Tumour Society (ENETS) and other bodies is that patients should have a triple phase CT chest abdomen and pelvis [2, 18]. While CT scans are recommended by ENETS, contrast enhanced magnetic resonance imaging (MRI) scans have a higher sensitivity for identifying liver metastases. MRI is regarded as the gold standard prior to consideration of any hepatic surgery as a minimal requirement [19]. MRI has demonstrated higher sensitivity for NELMs, compared to CT scans; contrast-enhanced CT scans demonstrate a sensitivity ranging from 41.7 to 77%, while contrast-enhanced MRI demonstrates a sensitivity range of 42.9–92.3% [20–24].

The standard MRI protocol contains the following sequences: axial T2, axial T1 in-phase and out-of-phase, fat-suppressed T2-weighted, diffusion weighted imaging (DWI) with corresponding apparent diffusion coefficient map, which includes both morphological and functional features of the lesion, and volumetric T1-weighted pre-contrast and dynamic volumetric post-extracellular contrast sequences, to contain at least an arterial and portal venous phase [25]. However, the European Society of Gastrointestinal and Abdominal Radiology has recommended use of liver-specific contrast agents, including gadobenate dimeglumine (Gd-BOPTA) and gadoxetic acid (Gd-EOB-DTPA, Primovist®) [26]. Studies have found that gadoxetic acid-enhanced MRI detected additional lesions, when compared to extracellular gadolinium-enhanced MRI scans [27•, 28, 29]. As with iodinated contrast media in CT, with extracellular gadolinium agents in MRI and liver-specific agents in MRI, NELMs typically show avid arterial phase enhancement. As they do not contain functioning hepatocytes, they do not take up liver-specific MRI contrast in the hepatobiliary phase and appear hypointense. This imaging feature is not specific to NELMs and is seen in other benign and malignant lesions. It is therefore vital that all imaging data are reviewed in conjunction with the hepatobiliary phase to avoid compromising on specificity when diagnosing NELMs.

Positron emission tomography (PET) for NETs is performed using different tracers and is usually fused with unenhanced CT (PET-CT). These tracers include, F-18-fluorodeoxyglucose (FDG) and somatostatin receptor PET tracers, like Ga-68-DOTANOC, Ga-68-DOTATOC, and Ga-68-DOTATATE [25, 30]. For detection of NELMs, Somatostatin receptor PET scans have high sensitivity [31, 32]. A combination of CT and SSTR PET scans has improved diagnostic accuracy when compared to any individual scan, therefore SSTR PET-CT imaging should be considered prior to all patients undergoing systemic therapy or local regional therapy for neuroendocrine tumours. FDG-PET-CT scans should be considered for all G3 NETs, NECs, and certain G2 NETs – Ki-67 > 10% [33–35]. Generally, patients being considered for liver surgery or liver directed therapy should undergo a CT chest, abdomen pelvis (with imaging of the liver pre-contrast and post-contrast in the arterial and venous phases) and an MRI of the liver, with consideration of a PET scan.

### Classification of Liver Metastases

Once imaging characterisation of NELMs has been undertaken as outlined above, a classification model can be applied. The most used model was proposed by Frilling et al. [36•]. This system characterises the spread of liver metastases into 1 of 3 distributions, see Table 1. Type I is a single metastasis of any size, Type II is an isolated metastatic bulk alongside smaller deposits with involvement of both lobes, and Type III is disseminated metastatic spread with both lobes always involved, or a single lesion of varying size with virtually no normal hepatic parenchyma [36•].

More recently, Mahuron and Singh have suggested a new approach relating to the management of liver metastases from NETs [37]. This new approach classifies liver metastases into four domains assessing tumour burden and ability for surgical interventions. However, this classification is a recent proposal and is yet to be evaluated or adopted by other societies at present.

**Table 1:**
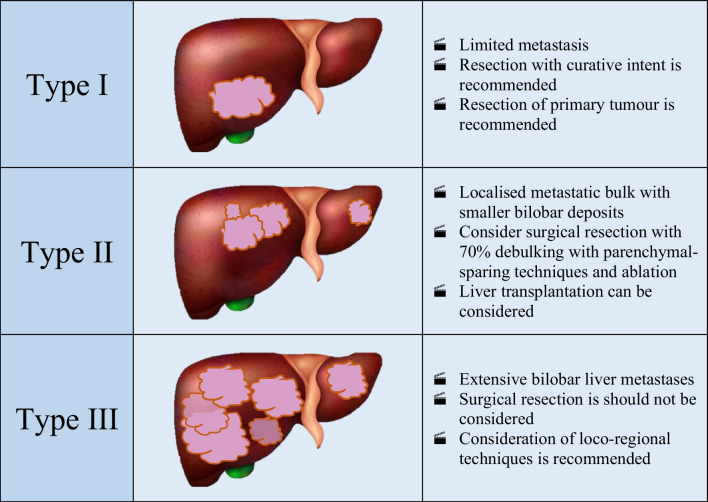
Classification system for neuroendocrine tumour liver metastases

- Type I patients have an isolated metastasis that can be completely cleared with hepatic debulking. Hepatic resection can be performed at the same time as Primary Tumour Removal (PTR) including in synchronous cases.
- Type II patients have multiple lesions spread across both lobes, however, 70% or more debulking can be achieved by utilising parenchymal sparing techniques and ablation. PTR can again be considered in this cohort of patients but may be delayed dependant on the extent of surgery needed.
- Type III patients have extensive, bilobar hepatic involvement. In these circumstances, greater than 70% debulking clearance cannot be achieved and therefore cytoreduction should not be considered surgically. These patients may be suitable for liver directed therapies such as transarterial embolization (TAE) or radioembolisation. PTR should be considered, as survival benefit may be increased even without liver directed interventions.

## Management of Liver Metastases

There are several different treatment options available for patients with liver metastases and numerous other factors need to be considered, including performance status, patient wishes, distribution of disease, presence of primary tumour in situ, grade of tumour and extent of extra-hepatic disease. It is imperative that discussions regarding management are held within a multidisciplinary team meeting and in consultation with the patient.

Whilst hepatic surgery is a well-recognised treatment option for patients with liver metastases there is no randomised control trial (RCT) comparing surgery to medical therapy. There are numerous publications of long-term survival in surgical cohorts which suggests excellent long-term survival. However, two retrospective comparative propensity matched cohort studies have not demonstrated any evidence of benefit of surgery with medical therapy compared to medical therapy alone [38•, 39]. The authors, however, recognise that there were limitations, including the small study cohorts, especially after propensity score matching [38•, 39]. In addition, both papers agree that despite not demonstrating any significant differences in survival, liver resection can be considered, in specific cohorts of patients [38•, 39].

## Patient Considerations for Liver Surgery

At initial work up and prior to consideration of surgery, nutritional status and performance status should be assessed, also a co-morbidity profile should be created. In patients with carcinoid syndrome, assessment for carcinoid heart disease should be performed at baseline initial work up. Patients with evidence of carcinoid heart disease may need heart valve surgery prior to liver resection since high right-sided pressure can complicate liver surgery [40]. For patients with pancreatic NET, gut hormone profile should be performed to confirm whether tumour is functional or non-functional.

In NETs of unknown primary tumour location, if the above imaging and biochemical work up with gut hormone profile and urinary 5-HIAA has not identified a primary site, then further indication of primary site may be determined from histological assessment. Immunohistochemical assessment to help determine the likely primary site can be performed including CDX-2 and TTF-1 stains [41]. There may be a role for MR enterography if a small bowel primary is suspected [42].

Regarding anaesthetic consideration for primary resection, generally patients with good performance status - Karnofsky > 70 and American Society of Anaesthesiologists (ASA) grade 1–2 - should be considered for surgery. Patients with ASA grade 4 are not generally suitable for surgical intervention apart from exceptional circumstances. Patients ASA grade 3 need careful discussion about likely tumour behaviour and whether surgery will improve overall survival (OS), especially in the era of peptide receptor radionuclide therapy (PRRT) and other therapeutic alternatives.

## Histological and Tumour Considerations for Surgery

For G1 and G2 NETs, surgery with curative intent should always be considered, even in the face of liver and lymph node metastasis [2]. For G1 tumours, the rate of progression is generally often rather indolent, however, for patients with G2 tumours this can be varied, and it is not unreasonable to have a period of observation prior to embarking on major surgery. For patients with G3 neuroendocrine tumours, surgical consideration can be undertaken in the context of metastatic disease; however, this has to be weighed carefully with ensuring that resection with curative intent can be achieved and also that there is a degree of tumour stability prior to considering surgery [43].

## Outcomes from Surgical Resection

To date, there have been no RCTs comparing surgery against non-surgical therapies. Despite this, hepatic resection is thought to be associated with the best long-term outcomes for NELMs [44]. 5-year OS can reach 60–80% among well-selected patients [45]. A systematic review comparing surgery to no surgical intervention, chemotherapy, and liver-directed therapies, found significantly improved OS for the surgical groups [46].

There are numerous studies looking at the outcomes of liver surgery with NETs, however, many of these are single centre studies. The largest published single centre series recently came from the Mayo Clinic Rochester with a cohort of 546 patients with NELMs from 2000 to 2020 [47]. 75% achieved complete resection, and 20% achieved > 90% reduction [47]. Gudmundsdottir et al. divided the time into 3 discrete segments (2000–2006, 2007–2013, 2014–2020): the 5-year OS was 71%, then 78%, and the final trimester 81%, demonstrating improved outcomes over time. The study found that the strongest predictor of OS was the Ki-67. Primary tumour site, number and size of hepatic lesions, and presence of distant metastases also affected survival [47]. A systematic review by Saxena et al. looked at survival outcomes across 24 papers on surgical resection of NELMs and the 5-year OS was 70.5% [48].

Historically, the target for debulking was 90% or more which was postulated by McEntee et al. in 1990 [49]. This was reinforced by a large series by Sarmiento et al., leading to the adoption of > 90% cutoff for resection [50]. However, in the last 10 years there are several studies that suggest achieving 70% debulking can offer very significant improvement in outcomes [43]. It is prudent to challenge the current threshold as only 20% of patients with NELMs are considered for cytoreductive surgery [43]. Multiple studies have suggested favourable outcomes with a 70% cut-off and several centres are advocating this approach [51]. These studies may confer an inherent bias as they are non-randomised, single-centre studies that compare their survival statistics to historical controls. Yet, in the face of a lack of RCTs, these studies form the foundation for the 70% debulking threshold.

Chambers et al. proposed that patients with NELMs may still obtain benefits with hepatic cytoreduction using a lower debulking threshold [51]. In a study with 66 patients undergoing cytoreduction, a 74% 5-year OS was reported [51]. This finding was further supported by Graff-Baker et al. [52]. A landmark study by Maxwell et al. demonstrated a survival benefit using a debulking threshold of 70%: 108 patients with NELMs underwent cytoreduction ranging from < 50% to > 90% debulking [53•]. They found that patients with > 70% debulking had improved OS, compared to those with < 70% debulking [53•]. An update study from the same institution reported outcomes for 188 patients, once again showing improved OS for patients with > 70% cytoreduction [54]. There was an improved progression-free survival (PFS) for patients that underwent > 90% versus 70–90% debulking, however, there was no statistically significant difference in OS between the two groups [54]. Another large single institution evaluated 189 patients with > 70% debulking threshold for NELMs; they reported a 5-year OS of 87% [55].

Laparoscopic resection is favoured over open surgical resection for NELMs when possible. A study compared the two surgical methods for resection and found statistically significant differences in total operative time, blood loss, transfusion, and duration of post-operative stay [56]. Laparoscopic resection provided shorter duration of surgery, with fewer transfusions required, and with shorter post-operative inpatient stay.

Regardless of surgical approach, nearly all patients will have recurrence of their hepatic disease [57]. A systematic review indicates that recurrence after resection ranges from 50 to 95%, with most occurring in the liver [48–52, 53•, 54, 55]. However, in selected patients, repeat hepatic resection of recurrent NELMs has demonstrated to be feasible and associated with good long-term survival outcomes [58].

Historically, NELM management with debulking aimed for R0, leading to major hepatectomy and loss of functional parenchyma [43]. However, multiple studies have documented equivalence in patient outcome with R0 versus R1/R2 resections [57, 59, 60]. Moreover, debulking surgery (R1/R2 resection) can improve the quality of life of patients for whom optimal medical management does not provide adequate control [60]. Uptake of parenchyma-sparing debulking (PSD) accepts the inevitability of recurrence and benefits the patient by minimising the volume of healthy liver tissue removed or damaged during the operation [53•]. Patients treated with PSD attained superior 5-year OS compared to using laparoscopic radio-frequency ablation (RFA) alone (72% vs. 57%) [61]. Furthermore, PSD is associated with low morbidity and mortality, with the literature suggesting morbidity rate of 20–30% [53•]. In the study by Maxwell et al., only 13% of patients experienced a major complication because of their debulking operation; there were no mortalities, no pancreatic fistulas requiring drainage, no bile duct injuries, nor hepatic abscesses noted [53•]. These results suggest these procedures may be performed safely and have the advantage of leaving more functional liver tissue intact [53•].

Patients with extensive liver metastases may not be considered for resection due to the risk of post-operative liver failure because of inadequate remaining functional parenchyma to support post-operative liver function [62, 63]. Post-operative liver failure is directly associated with the volume of the future liver remnant (FLR) [64]. This can be mitigated through the induction of parenchymal hypertrophy using portal vein embolisation (PVE) [62]. Occlusion of the portal vein supplying the hepatic lobe for resection induces contralateral lobar hypertrophy. There is no consensus on the most appropriate waiting time between PVE and liver resection, however, one meta-analysis showed an average interval of 29 days [65]. A single-centre, retrospective study demonstrated that the FLR function (as assessed by hepatic scintigraphy) exceeded the FLR volume gain after PVE [66]. This provides evidence that PVE provides functional gain and is not only limited to volume gain. The combined role of pre-operative loco-regional therapies with PVE for NELMs has not yet been assessed.

A meta-analysis compared PVE with dual vein embolisation (DVE) for FLR in patients undergoing resection [67]. DVE involves simultaneous embolisation of the portal vein and the hepatic veins which induces hypertrophy of the FLR. Bell et al. found that the percentage increase in hypertrophy was 66% in the DVE compared to 27% in the patients who received PVE [67]. Moreover, rates of complications were lower in the DVE group, although this was not statistically significant. Perioperative mortality was statistically significantly lower in the DVE group.

Associated liver partition and portal vein ligation for staged hepatectomy (ALPPS) aims to offer a more rapid and increased hypertrophy response, when compared to PVE. The International ALPPS Registry was retrospectively reviewed to look at the 24 patients who received ALPPS for NELMs, from 2010 to 2017 [68]. The median time between stage 1 ALPPS and stage 2 was 11 days (8–21 days) and there was a 66.7% increase in standardised FLR (45.8 − 103.3%) [68]. While ALPPS demonstrates a greater degree of hypertrophy, it is associated with increased morbidity and mortality, when compared to PVE. A comparison of 51 patients who underwent PVE with 12 patients undergoing complete ALPPS found that: 18% and 30% of patients experienced severe post-resection complications and the 90-day mortality was 2% and 25%, respectively [66]. These values must be taken in the context of the small sample size; however, they indicate the risks associated with ALPPS.

Early recurrence after complete cytoreduction, defined as recurrence within 3 years, typically represents a poor prognosis. For intrahepatic recurrence, pancreatic NET, primary lymph node tumour metastasis, and microscopic positive surgical margin were independent associations on multivariate analysis [69]. Pancreatic NETs have a lower 5-year OS (30-60%) compared to intestinal NETs (60-90%) [70–72].

Management of NECs proves challenging due to the aggressive nature of the disease, combined with a paucity of trials comparing treatments. A study of 32 patients with NECs and well differentiated NETs (G3 NETs) looked at the OS and prognostic factors [73]. Galleberg et al. found that median OS after resection or RFA of liver metastases secondary to NECs was 35.9 months, with a 5-year OS of 43% [73]. Prognostic factors that were shown to improve OS with statistical significance were a Ki-67 < 55% and receiving adjuvant chemotherapy during surgical or ablative interventions [73].

### Liver Transplantation

Due to the relative rarity of NETs and low proportion of liver transplantations (LT) carried out for NELMs, there are no RCTs comparing LT with surgical excision or other NELM management [74]. Despite this, LT has expanded significantly within the field of NETs over the last 20 years; the European Liver Transplant Registry (ELTR) has recorded over 500 patients having undergone liver transplantation for NETs. There are several considerations regarding when to consider liver transplantation. Firstly, weighing the risks and benefits of transplantation, considering survival on immunosuppression and the risk of developing disease recurrence. Secondly, there is the continuous issue of organ donor scarcity [75, 76].

Several liver transplantation programs have published criteria for the selection of patients suitable for transplantation, they are all based on the Milan criteria with some variations from this approach [77]. The Milan criteria, developed in 1995, was updated in 2016 to reflect the findings from Mazzaferro et al. [78•]. The United Network for Organ Sharing (UNOS) criteria builds on the Milan criteria regarding tumour recurrence with the aim to reduce the risk of transplantation waiting list dropout [75], see Table 2.

**Table 2:** Selection criteria for orthotopic liver transplantation (OLT) for liver metastasis of a neuroendocrine tumour

	Milan criteria 2016 [78•]	UNOS guidelines 2017 [79]	ENETS guidelines 2016 [2]
Histology grade	G1–G2	G1–G2	G1–G2
Primary tumour site	Portal system drainage	Portal system drainage	NA
Tumour involvement	< 50% of the liver volume	< 50% of the liver volume	NA
Time interval of stable disease between primary tumor resection to liver transplantation	Resection of primary tumor and all extra-hepatic tumor deposits and stable disease > 6 months	Resection of primary malignancy and extra-hepatic disease without any evidence of recurrence > 6 months	NA
Recipient age	< 60 years	< 60 years	NA
Other	Extended Milan criteria < 70 years	GEP origin NELM restricted to the liver, bi-lobar, not amenable to resection Negative meta workup	Functional NETs and diffuse liver disease, early refractory to multiple systemic treatment; exclusion of extrahepatic disease; low bilirubin; carcinoid syndrome or functional NETs

NA not applicable; GEP gastroenteropancreatic; NET neuroendocrine tumour; NELM neuroendocrine liver metastasis.

Mazzaferro et al. demonstrated a 5-year survival post-transplant of 97.2% (*n* = 42), compared to the control group of 50.9% (*n* = 46) [78•]. However, there is a possible selection bias within this study: the transplant group was younger than the control, (median age 40.5 years vs. 55 years) and the transplant group received more loco-regional therapy prior to intervention (40.5% vs. 21.7%) [78•]. These results are contextualised by the stringent patient selection criteria: only 88 patients met the Milan criteria of the 280 patients considered for LT. Due to patient wishes or medical contra-indication, only 42 went on for LT, 15% of the original cohort [78•]. Furthermore, retrospective application of the Milan criteria to a cohort from the ELTR, the 5-year OS increased from 59 to 79%. But this was at the expense of excluding 64% of all transplanted patients [80]. The most promising finding from the study run by Mazzaferro et al. was the improved benefit of OLT over time. The survival gains in favour of transplantation, compared to no transplantation, were 6.8 months at 5 years and 38.4 months at 10 years [78•].

One factor that is thought to play a crucial role in the success of the study in Milan, is the delay of more than 6 months criterion. This selected inclusion criterion suggests that patients with stable disease with no symptoms will see true benefit from LT, compared to medically refractory disease [81]. Further prospective studies will be required to demonstrate the causative relationship with improved 5-year OS and the > 6-month disease stable inclusion criteria.

The large studies in NELM liver transplantation did not include the post-transplant immunosuppressive therapy used, except for Mazzaferro et al. who reported on 41 patients [78•]. They described the tapering of steroids in the third month post-transplant and the initiation of calcineurin inhibitor monotherapy (tacrolimus and cyclosporine in 81% and 19% of patients, respectively) [78•]. Most liver transplant centres have immunosuppressive regimes that include calcineurin inhibitors, antimetabolites, and steroids [82]. Everolimus was approved for prevention of graft rejection in OLT in October 2012 in Europe [83]. It may be an alternative for transplant patients with renal impairment; this is particularly useful especially in cases of calcineurin inhibitor nephrotoxicity; s study found that 18% of LT recipients developed renal impairment within 5 years of LT [83]. Therefore, Everolimus should be considered in patients at risk of renal impairment.

Recurrence after LT ranged from 31.3 to 56.8% based on aggregated multicentre data [84]. A study looking at LT for NELMs used a propensity score to match patients with hepatocellular carcinoma (HCC) and cholangiocarcinoma to compare disease recurrence and 5-year OS after LT. While disease recurrence rates were comparatively higher: NET recurrence 34%, HCC recurrence 8% and cholangiocarcinoma 19.6%, there was no statistically significant difference in 5-year OS: 75.4%, 79.9%, and 70.4%, respectively [74]. However, propensity scores are limited by an inability to avoid unmeasured confounding factors and are inefficient when considering small sample sizes [85].

The recurrence pattern differs in LT and liver resection. Maspero et al. looked at the Milan group analysis and found that in the patients with recurrent disease, transplanted patients experienced more multisite recurrence (48% vs. 12%) but had longer time to recurrence (6.5 years vs. 2 years) [86•]. Moreover, the recurrences in LT patients were less likely to occur in the liver (8% vs. 88%) than resected patients. There was no statistically significant difference in post-recurrence survival at 3-years or at 5-years [86•].

## Loco-regional Therapies

Patients who do not meet the criteria for liver resection or transplantation due to performance status or extensive liver disease can undergo loco-regional therapies [87]. Given that the degree and frequency of involvement, control liver disease by loco-regional therapies can be important for prognosis [44]. Selection of appropriate therapy and combination with systemic therapy is based on individual patient features and clinical judgement due to the absence of large comparative trials for liver-directed therapies [2].

### Ablative Therapies

Radiofrequency ablation (RFA) – RFA is one of the oldest ablative techniques and has the potential to be used in conjunction with other therapies [77]. Moreover, RFA can be used multiple times on the same lesion [88]. RFA has been shown to provide greater tumour control when done laparoscopically or with open surgery, when compared to percutaneous methods; this holds true irrespective of the size of the lesion [89]. The largest series using RFA was conducted by Mazzaglia et al. and looked at 452 lesions managed laparoscopically in 63 patients [90]. The study found that median survival was favourable in patients with lesion < 3 cm. Tumours < 3 cm and at least 1 cm circumferential post-ablation margin also demonstrated the lowest local recurrence rate [91]. A systematic review of RFA for NELMs demonstrated excellent symptom control; combined with its low morbidity and mortality rates [92], RFA therefore offers itself as an option for symptom palliation and can help to reduce the dose of concurrent somatostatin analogue medication [93].

Microwave Ablation (MWA) – MWA makes use of electromagnetic microwaves for thermal ablation of LMs [94]. MWA has been used extensively for HCC and colorectal carcinoma, however there is limited study of its use in NELMs [95]. The studies that have been carried out so far suggest MWA may be superior to RFA in management of larger NELMs [96] and it is well established that MWA produces larger ablation zones and is less vulnerable to the “heat-sink” effect [97]. Pickens et al. reviewed a cohort of 50 patients in a single-centre retrospective study looking at curative and cytoreductive MWA for NELMs [98]. They found that 5-year survival was 70% for those who underwent MWA for curative intent and 69% for patients undergoing MWA for cytoreduction [98].

Irreversible Electroporation (IRE) – IRE works by delivering pulses of electrical current of high voltage between electrodes to induce non-thermal apoptosis [99]. The main advantage of IRE is that it spares the collagenous structures supporting the biliary ducts and main liver vessels and should not cause thermal damage to these structures. As a result, it is particularly indicated for the treatment of very central liver metastases [100]. The use of IRE for NELMs is limited, however, one single-centre study found a complete ablation rate of 88% for hepatic tumours with IRE [101]. The study displayed a statistically significant reduction in recurrence of hepatic tumours with the use of more probes per tumour combined with more sets of pulses [101].

### Intra-arterial Therapies

Transarterial embolisation (TAE) involves administering embolic beads into the artery supplying the NELM, thereby inducing selective ischaemic necrosis to the lesion [4, 102]. This method is also known colloquially as “bland” embolisation [87]. This contrasts with transarterial chemoembolization (TACE) which uses cytotoxic chemotherapeutic agents either loaded onto embolic beads or mixed with lipiodol as an emulsion [102]. The most used chemotherapy agents used are streptozotocin or doxorubicin [102]. While TACE is a more recent technique, a meta-analysis, including 504 patients managed with TAE or TACE, revealed no difference in median OS, symptom improvement, morbidity, or mortality [103]. There was no statistically significant difference in OS: TAE OS ranged from 21 to 65 months, compared to TACE range of 25–68 months [103]. Following treatment, it is recommended that patients are kept overnight to manage post-embolisation syndrome [88]. This is characterised by fever, nausea and vomiting, abdominal pain, and transaminitis [88]. This is a common complication, however, with repeat TACE sessions, the symptoms decrease over time [10, 104].

Selective internal radiation therapy (SIRT) – SIRT is an alternative intra-arterial therapy that involves the delivery of particles loaded with a radioactive substance (most commonly Yttrium-90) to NELMs [104]. Unlike TAE/TACE, it does not rely on an embolic effect, but cell death is induced by local delivery of high energy β radiation. Two meta-analyses, including 19 and 11 studies, found the median OS after SIRT was 28 months and 32 months [106, 107]. In a retrospective study of 72 patients, SIRT yielded a better 3-year survival rate than TACE (88% vs. 53%) in patients with a Ki-67 > 3% [108].

In a study by Chen et al., 50 patients received TACE, 64 received transarterial radio-embolisation (TARE), and 41 received TAE. In this cohort, median hepatic PFS was 8.1, 15.7, and 15.0 months, with no statistically significant difference [109]. Another study showed that TACE had an improved OS than TARE (16.8–81.9 vs. 14.5–66.8 months) [110]. However, there was no difference in 3-month tumour response (*p* = 0.10) or response after 3 months (*p* = 0.99) [110]. While SIRT may not display any superiority over TAE or TACE with respect to OS, it has lower rates of, and milder, post-embolisation syndrome, leading to better post-procedural quality of life [111–113]. SIRT can also be considered after failed TAE or TACE [88]. Importantly, due to its lack of significant embolic effect, SIRT is likely to be safer than TAE or TACE for patients at high risk for biliary complications; these include Sphincter of Oddi incompetency and biliary-enteric anastomoses [114]. However, there is a risk of chronic liver disease following treatment with radioembolization, therefore, careful patient selection to minimize this risk and also consideration of the previous therapies patients have received should be considered [115].

SIRT with holmium-166 confers potential advantages over yttrium-90 products as the work-up procedure may be more accurate [115]. Moreover, holmium SIRT can be combined safely with PRRT [115]. The NETTER-1 study showed worse PFS with PRRT in patients with bulky liver disease, defined as tumours larger than 3 cm, compared to those without [117]. Therefore, SIRT may play a role in patients with bulky liver dominant disease. Given the time to symptomatic improvement with PRRT, SIRT may also prove more beneficial for more rapid symptom control. The exact role for SIRT in treatment of NELM will require further evidence to establish.

The ENETS recommend SST analogues prior all locoregional therapy in patients with functioning tumours to prevent carcinoid crisis [2]. However, many studies have failed to detect a reduction in carcinoid crisis or haemodynamic instability in patients receiving SST analogues [118–120]. Further RCTs are required to elucidate the definitive efficacy of SST analogues in reducing post-procedure carcinoid crises.

## Systemic Therapies

Full discussion of the systemic therapy options available for patients with NELMs is beyond the scope of this article and is covered in ENETs guidance papers [2]. We have briefly highlighted the therapies the systemic therapies currently available.

NELMs often requires systemic therapy to manage tumour bulk and symptoms caused by liver involvement, as well as for control of distant metastases [93]. SST analogues, like Octreotide and Lantreotide, are used first-line for functional tumours [121–123]. . They are also recommended for control of tumour growth as evidenced from the PROMID and CLARINET studies [124, 125].

The use of chemotherapy in NELMs is dependent on functional activity and grading of the tumour. Location of primary tumours can also be an important factor for selection of combination therapies. For example, combination of Streptozotocin and 5-Flurouracil or Doxorubicin in mid-gut NETs yielded response rates of 10–15% [57, 126, 127], whereas in NETs originating in the pancreas, response rates reached 39% with improved OS [128].

PRRT involves the intravenous injection of radionuclides such as Yttrium-90 or Lutetium-177 which are bound to SST analogues [87, 102], . This complex binds to the SSTRs that are highly expressed on sites of neuroendocrine tumour metastases and the agent induces necrosis through release of β radiation [129]. PRRT is indicated in well differentiated progressive GEP NETs with significant SSTR expression [102].

Yalchin et al. demonstrated an OS of 33.5 months and PFS of 28.5 months in a cohort of 133 patients with midgut NELMs [130]. PFS was improved in patients with greater number of PRRT cycles, as well as in patients who had resection of NELMs prior to PRRT. Thus, patients may benefit from using PRRT following resection of NELMs [130, 131]. Intra-arterial PRRT has been compared to intravenous PRRT and demonstrated an improvement in OS [132]. However, the termination of a trial in 2021 after analysis of the first 10 patients raises concerns over the safety of intra-arterial PRRT [133]. PRRT has known adverse reactions, commonly, toxicity to kidneys, bone marrow, and liver [134]. Another concerning adverse effect is myeloid neoplasms. Goncalves et al. found that 25 out of 521 patients (4.8%) from a single institution developed myeloid neoplasms after PRRT [135].

## Therapeutic Algorithm for NELMs

**Fig. 1:**
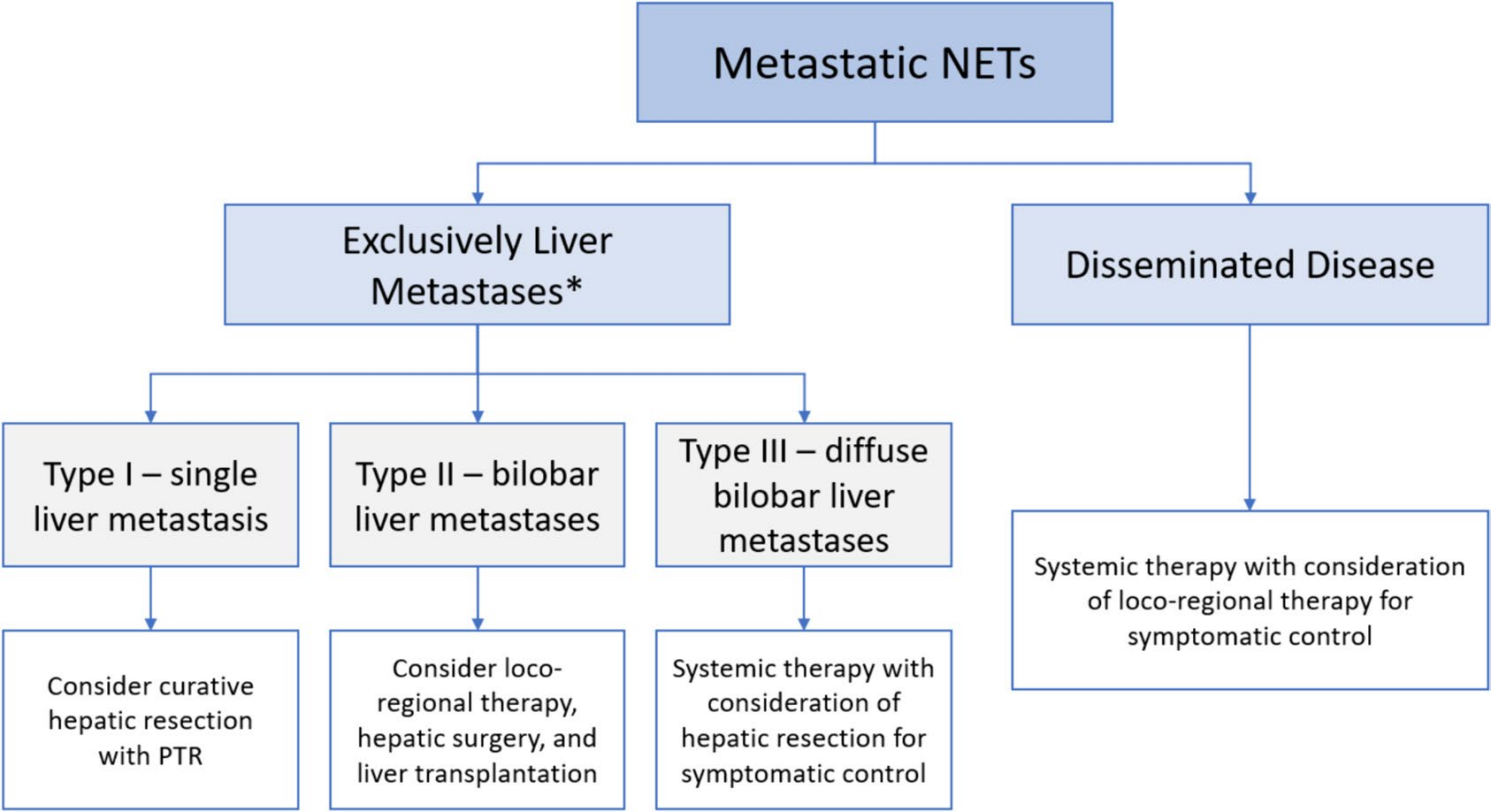
The proposed algorithm for approach to management of liver metastases. PTR = primary tumour resection; NET = neuroendocrine tumour. *Can consider liver resection if small extra-hepatic disease may be resected at time of surgery or other small sites of disease not felt to have significant impact on long term survival

For type II liver metastases, careful assessment of the distribution is required to assess whether surgery can be undertaken and whether volume manipulation of functional liver volume needs to occur. Also, in selected cases, consideration of liver transplantation can be considered. There may be a role of debulking liver surgery for type III cases, however, this is purely for symptomatic purposes. Figure 1 proposes a general approach to management options for NET liver metastases.

## Conclusions and Further work

NELMs are common in patients with NETs; their presence and degree of tumour burden are key prognostic factors. Moreover, NELMs can lead to significant morbidity that worsens quality of life. Therefore, optimal management of NELMs requires careful consideration in a multidisciplinary team and always with patient involvement.

Primary tumour resection with hepatic resection of NELMs should always be considered. Liver-directed therapies and liver transplantation can be considered for patients with unresectable liver metastases. Systemic therapies play a role in managing tumour burden and symptoms secondary to NELMs.

The heterogeneity of NETs makes research challenging due to variation in baseline disease, comorbidity, and prior management. Despite this, more work is needed to reduce disease burden and improve quality of life. Prospective RCTs comparing medical and surgical management options will offer stronger evidence for selection of appropriate treatments. Furthermore, novel therapies, like IRE have yet to be used in multi-centre studies for NELMs.

## Data Availability

No datasets were generated or analysed during the current study.
